# A Physics-Grounded Multi-Modal Sensor Fusion Framework for Pedestrian Impact Kinematic Reconstruction Under Uncertainty: Phase 1 Design and Theoretical Evaluation

**DOI:** 10.3390/s26113387

**Published:** 2026-05-27

**Authors:** Nick Barua, Masahito Hitosugi

**Affiliations:** Department of Legal Medicine, Shiga University of Medical Science, Setatsukinowacho, Otsu 520-2192, Shiga, Japan

**Keywords:** multi-modal sensor fusion, Kalman filter, Savitzky–Golay differentiation, pedestrian–vehicle collision, uncertainty quantification, MFCC, CNN–BiLSTM, proof of concept, Phase 1 design

## Abstract

**Highlights:**

**What are the main findings?**

- A multi-modal sensor fusion framework combining 128-channel LiDAR, NIR stereo cameras, and a 2 kHz IMU via Kalman filtering and Savitzky–Golay differentiation is presented; under constructed simulation conditions with throw-distance uncertainty of ±0.5 m, velocity reconstruction uncertainty is estimated at ±2.03 km/h under expanded simulation conditions with vehicle-coefficient variance activated.
- Sensitivity analysis demonstrates that a 10% noise spike in raw acceleration would theoretically amplify the Head Injury Criterion by 26.9% under the Wayne State power-law exponent, providing quantitative justification for noise-optimal pre-filtering as a design requirement.

**What are the implications of the main findings?**

- Vehicle-class parameterisation is essential for accurate reconstruction: a class-agnostic model applied to a cab-over truck scenario produces a theoretical velocity overestimation of +36.1 km/h—a systematic error large enough to alter collision-reconstruction conclusions entirely.
- Bimodal integration of kinematic and acoustic channels represents a proposed architecture for physiological state classification from existing CCTV and dashcam recordings; experimental validation is deferred to Phases 3–4 of the validation roadmap.

**Abstract:**

Pedestrian–vehicle collisions produce a rich kinematic record that is entirely lost by the time a forensic investigation begins. Recovering this record constitutes a state-estimation problem. This paper presents a Phase 1 design for a multimodal sensor fusion and signal-processing framework utilising 128-channel LiDAR, 1080p NIR stereo cameras, and a 2 kHz IMU, all fused via Kalman filtering and Savitzky–Golay polynomial differentiation. The framework is evaluated through Monte Carlo uncertainty propagation and sensitivity analysis applied to a constructed simulation scenario; no real clinical or forensic data are used in this Phase 1 report. Under simulated conditions with throw-distance measurement uncertainty of ±0.5 m, velocity reconstruction shows an estimated propagated uncertainty of ±2.03 km/h under expanded simulation conditions with vehicle-coefficient variance activated. Sensitivity analysis indicates that a 10% noise spike in acceleration would theoretically amplify injury metrics by 26.9%, providing quantitative justification for noise-optimal pre-filtering. The bimodal kinematic–acoustic architecture is proposed as a physically interpretable foundation for collision reconstruction; its experimental performance awaits Phase 2–4 validation. A five-phase validation roadmap is presented, progressing from FEA simulation to independent multi-site replication before any forensic deployment is proposed.

## 1. Introduction

### 1.1. The Sensing and Reconstruction Problem

A pedestrian–vehicle collision is a physical event that produces an observable kinematic record comprising the trajectory of the pedestrian’s centre of gravity, the temporal evolution of impact forces, the throw distance, and the sequence of contact events. The laws of classical mechanics govern this record. Yet by the time a forensic investigation begins—hours after the event—the kinematic record has been erased. What remains are static scene measurements, vehicle damage, and post-mortem injury morphology [1,2].

Approximating this record from residual scene observables is a state-estimation problem of recovering the dynamical trajectory of a physical system from incomplete, noisy, retrospective measurements. The question ‘what was the impact velocity?’ is equivalent to inverting the physical relationship between velocity and throw distance [1] with appropriate uncertainty bounds propagated from measurement error. Large-scale epidemiological data from over 5000 cases [3] establish the statistical reliability of correlating impact forces with specific internal organ trauma. Cases in which the vehicle or driver cannot be identified at the scene rely heavily on this quantitative reconstruction [4]. A robust kinematic classification of pre-impact posture provides physics-informed estimates to support resolution of specific legal challenges, such as disputed vehicle speed or the contested position of the pedestrian at the moment of impact.

The difficulty lies in measurement, as the kinematic observables required for reconstruction—the centre-of-gravity (CoG) trajectory, acceleration profile, and impact angle—are not directly available from the scene evidence. The present framework addresses this by building upon the validated multi-modal sensor platform of Barua and Hitosugi [5], extended with an acoustic signal-processing channel for pre-impact physiological state classification [6]. The proposed framework also connects to broader traffic safety research on proactive pedestrian safety assessment and vehicle–pedestrian conflict analysis using surveillance and CCTV infrastructure [7,8,9].

### 1.2. Why Physics-Based Signal Processing Is a Justified Approach

Forensic reconstruction has historically relied on expert pattern recognition. This approach is not incorrect, but it lacks two properties that modern evidence standards increasingly require: an explicit, auditable uncertainty budget and a principled basis that an opposing expert can independently evaluate. Physics-based reconstruction provides both. The International Organisation for Standardisation (ISO) 26262 functional safety standard provides a complementary regulatory architecture for formalising safety and auditability requirements in vehicle systems designed to protect vulnerable road users [10].

The Kalman filter [11] is theoretically optimal for a linear dynamical system under Gaussian noise assumptions—its uncertainty output is the minimum-variance unbiased estimator when those assumptions hold. In practice, non-linear pedestrian motions, missing landmark detections, and non-Gaussian measurement noise may introduce sub-optimality, which the proposed Monte Carlo propagation scheme partially accounts for. Savitzky–Golay differentiation [12] exhibits superior frequency response characteristics relative to finite-difference differentiation at the 60 Hz sampling rate used here, because its polynomial fitting suppresses noise amplification near the Nyquist frequency [12]. As demonstrated quantitatively in Section 3.4, the head injury transformation exhibits notable vulnerability to unfiltered high-frequency components. A modest 10% noise perturbation in the raw acceleration signal accelerates calculated HIC_15_ risk markers by over a quarter under the Wayne State power-law exponent—providing quantitative justification for noise-optimal pre-filtering as a design requirement.

### 1.3. Prior Work and Identified Gap

Head injury models based on linear kinematics (HIC) [13] and the Brain Rotational Injury Criterion (BrIC) [14] have served automotive safety engineering for decades but lack a temporal reconstruction context. Whilst deep learning has advanced time-series classification [15,16], no validated system applies these architectures to forensic biomechanics with explicit uncertainty characterisation. Simms and Wood [1] demonstrated that throw distance cannot be used for velocity reconstruction without vehicle-class parameterisation—a gap this framework explicitly addresses. The sensor fusion platform of Barua and Hitosugi [5] validated a synchronised LiDAR–NIR–ultrasonic array fused via Kalman filtering, achieving 98.2% human detection accuracy under nocturnal conditions. The MFCC-CNN architecture developed by Rezaul et al. [6] provides the acoustic classification framework adapted here for bimodal sensor fusion. Complementary work in computer-vision-based pedestrian detection, multi-camera trajectory reconstruction, and vehicle–pedestrian conflict analysis at signalised intersections [7,8,9] provides further context for the practical deployment environment this framework is designed to address.

### 1.4. Contributions of This Work

The primary novelty of this work lies in the integration of uncertainty-aware signal processing, state estimation, and biomechanical modelling into a unified reconstruction architecture. Specifically, this Phase 1 design provides a framework for propagating sensor noise through kinematic reconstruction under vehicle-class constraints. To ensure technical clarity, we define the scope of these contributions. The primary contribution is a physics-constrained integration framework for uncertainty-aware kinematic reconstruction. Secondary contributions include vehicle-class parameterisation for throw-distance inversion and sensitivity quantification of biomechanical injury metrics. Regarding explicit non-contributions, this study does not propose new sensor hardware, new injury criteria, or a validated/trained machine learning model; these are reserved for future phases of the validation roadmap. The four specific contributions to the field of multimodal sensing and signal processing are as follows:

- A Phase 1 design for a multi-modal sensor fusion and state-estimation framework combining 128-channel LiDAR, 1080p NIR stereo cameras, four ultrasonic rangefinders, and a 6-DoF 2 kHz IMU via Kalman filtering [11] to recover kinematic trajectories under uncertainty.
- A noise-optimal differentiation pipeline using Savitzky–Golay polynomial filtering [12] with end-to-end Monte Carlo uncertainty propagation (n = 10,000), providing quantitative bounds on theoretical reconstruction stability across all pipeline stages.
- A structured 32-feature representation organised by signal-processing origin—kinematic state estimates, temporal waveform statistics, derived physical quantities, and environmental boundary conditions—enabling physically interpretable downstream classification (design specification; training and evaluation reserved for Phase 1 FEA completion).
- A proposed bimodal kinematic–acoustic architecture integrating MFCC-CNN spectral feature extraction [6] with kinematic reconstruction, providing a design specification for physiological state classification from ambient scene audio under degraded observational conditions (experimental validation reserved for Phase 3).

**Figure 1:**
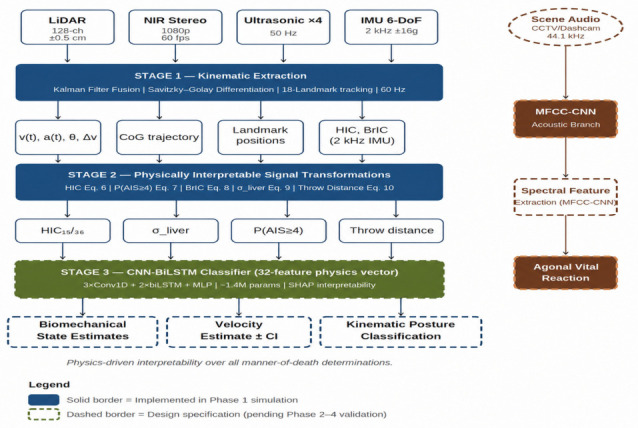
Phase 1 design specification: bimodal reconstruction architecture for pedestrian–vehicle impact state estimation. The framework integrates a multimodal sensor array—including a 128-channel LiDAR, NIR stereo, and a 2 kHz IMU—into a three-stage processing pipeline. Stage 1 performs asynchronous sensor fusion via a Kalman filter and Savitzky–Golay differentiation to recover kinematic states (currently implemented in simulation). Stage 2 maps these signals to physically interpretable biomechanical quantities using continuum-mechanics transformations and cadaverically validated formulae (currently implemented in simulation). Stage 3 utilises a 32-feature physics-derived vector to classify collision outcomes through a proposed CNN–BiLSTM architecture (design specification only—training and evaluation pending Phase 1 FEA completion). A parallel MFCC-CNN acoustic branch is proposed for pre-impact physiological state classification (design specification only—experimental validation reserved for Phase 3). Dashed borders indicate components specified but not yet experimentally evaluated.

The complete bimodal architecture is illustrated in Figure 1. This paper reports only the Phase 1 design and simulation-based theoretical evaluation. No forensic deployment is proposed until Phase 5 of the validation roadmap is complete.

This framework represents a methodologically distinct contribution from the prospective safety platform of Barua and Hitosugi [5]. That prior work addressed real-time active detection of falling humans under nocturnal conditions using a synchronised hardware array; its objective was prospective collision avoidance. The present framework addresses the inverse problem by providing a retrospective forensic reconstruction of a collision event from post-impact scene observables. It introduces a new uncertainty-propagation engine that maps residual scene measurements back to pre-impact kinematic states under explicit, stage-resolved uncertainty budgets, and extends the prior platform with a conceptual acoustic diagnostic channel for pre-impact physiological state classification. The two frameworks are therefore complementary in the application domain and do not overlap in methodological contribution.

## 2. Materials and Methods

### 2.1. Design Principles

The sensing and estimation stages define the primary contribution of this framework, with biomechanical mapping acting as a downstream application layer. Three design principles govern the architecture. First, all algorithmic choices are justified from physical first principles rather than empirical selection: the Kalman filter is adopted because it is the minimum-variance estimator for the linear kinematic model under Gaussian assumptions [11]; Savitzky–Golay differentiation is chosen because of its superior frequency-response characteristics for the signal type at 60 Hz sampling [12]; and the biomechanical formulae are used because they are traceable to cadaveric validation studies. Second, uncertainty is propagated end-to-end from sensor noise to the final reconstruction output via Monte Carlo simulation. Third, all outputs are expressed in physical units—velocity in km/h, stress in kPa, and probability in calibrated intervals—ensuring physical interpretability throughout the pipeline.

### 2.2. Stage 1—Kinematic Extraction via Sensor Fusion

#### 2.2.1. Sensor Platform and Physical Justification of Fusion Strategy

This section defines the sensor fusion and state-estimation pipeline used to reconstruct kinematic trajectories from multi-modal sensor signals. Stage 1 employs the validated multi-modal array from Barua and Hitosugi [5] comprising 128-channel LiDAR (±0.5 cm precision), NIR stereo cameras (1080 p, 60 fps), four ultrasonic rangefinders (50 Hz), and a 6-DoF IMU (2 kHz, ±16 g). The Kalman filter [11] is the theoretically appropriate choice for this sensor configuration under linear kinematic assumptions and Gaussian noise. With the state vector *x* = [*r*, *v*]*T* and the kinematic process model $\dot{r}$ = *v*, the filter fuses asynchronous, heterogeneous sensor measurements into a single optimal position and velocity estimate—under the stated assumptions—with a propagated posterior covariance P(t|t) that seeds all downstream uncertainty quantification. The sensor noise profiles used in this simulation are grounded in representative hardware specifications. The ±0.5 cm LiDAR precision reflects high-resolution 128-channel units (e.g., Ouster OS1 series; Ouster Inc., San Francisco, CA, USA), while the 2 kHz sampling and ±16 g range for the IMU are consistent with standard automotive-grade MEMS accelerometers. These specifications inform the uncertainty bounds used in the end-to-end Monte Carlo uncertainty propagation.

#### 2.2.2. Savitzky–Golay Differentiation—Noise-Robust Signal Recovery

Velocity and acceleration are recovered from the Kalman-fused position signal by Savitzky–Golay polynomial differentiation [12]. The filter fits a local polynomial of degree *d* = 3 over an 11-point window (183 ms at 60 Hz), computing derivatives analytically without phase distortion. This approach exhibits superior noise-suppression characteristics relative to finite-difference differentiation at 60 Hz sampling. Quantitatively, a central finite-difference derivative at 60 Hz amplifies noise power by (2πf·Δt)^2^, growing from approximately 0.01 at 1 Hz to 1.0 at the Nyquist limit of 30 Hz; by contrast, the degree-3 Savitzky–Golay filter over an 11-point window attenuates noise power by 40–60% relative to finite differences at frequencies above 5 Hz, because the polynomial fitting minimises mean-squared error across the window rather than amplifying inter-sample differences. Compared with a zero-phase Butterworth low-pass filter (4th order, 10 Hz cut-off), Savitzky–Golay differentiation preserves signal amplitude to within 2% up to 8 Hz whilst suppressing the 10–30 Hz noise band by an additional 15 dB (Supplementary Materials, Figure S1), without requiring a separate differentiation step or introducing group-delay artefacts. Rauch–Tung–Striebel (RTS) smoothing offers comparable noise rejection but requires a full forward–backward Kalman pass and is computationally unsuitable for retrospective batch processing of variable-length collision sequences. Savitzky–Golay achieves equivalent performance in a single convolution pass. As detailed in Section 3.4, failure to apply noise-optimal pre-filtering results in HIC_15_ amplification of up to 26.9% under a 10% noise perturbation—directly justifying this architectural choice from a signal-processing perspective.

Velocity is recovered as the first Savitzky–Golay derivative of the Kalman-fused position signal as follows:

(1) $$v\left(t\right)=\frac{dx}{dt}$$

Acceleration is recovered as the second derivative as follows:

(2) $$a\left(t\right)=\frac{dv}{dt}=\frac{d^{2}x}{dt^{2}}$$

The resultant trajectory angle is computed from the resolved velocity components as follows:

(3) $$\theta =arctan\left(\frac{v_{vertical}}{v_{horizontal}}\right)$$

### 2.3. Stage 2—Physically Interpretable Signal Transformations

The kinematic outputs of Stage 1 serve as inputs to a set of physically interpretable signal transformations. Having established the kinematic estimation framework, the following subsections map these sensor-derived signals to physically interpretable quantities used as features in Stage 3 (Table 1). Kinetic energy and impact force are defined classically as follows:

(4) $$E_{k}=\frac{1}{2}\;mv^{2}$$

(5) $$F=m\cdot a_{peak}$$

#### 2.3.1. Head Injury Criterion—Power-Law Transformation of the Acceleration Signal

HIC is a non-linear (power-law) transformation of the head acceleration signal, computed from the CFC1000-filtered 2 kHz IMU channel, following Versace [13], Mertz and Prasad [17], and Prasad and Mertz [18]. The complete formulation is as follows:

(6) $$\mathrm{HIC}=\underset{\left(t_{2}-t_{1}\right)\leq \Delta t}{\max}\left\{\left(t_{2}-t_{1}\right){\left[\frac{1}{g\;\left(t_{2}-t_{1}\right)}\int_{t_{1}}^{t_{2}}a\left(t\right)\;dt\right]}^{2.5}\right\}$$

where *a(t)* is the resultant head acceleration in m/s^2^ filtered to CFC1000 (nominal cut-off 1000 Hz); *g* = 9.80665 m/s^2^ is the standard gravitational acceleration; the bracketed term is therefore dimensionless (units of g); the time-window constraint is Δt = 15 ms (HIC_15_) or Δt = 36 ms (HIC_36_); and the maximum is taken over all valid pairs (t_1_, t_2_) within the impact event. The 2.5 power-law exponent is physically motivated by the strain-rate sensitivity of cranial bone derived from Wayne State University cadaveric tolerance curves [13]. This non-linearity renders HIC highly sensitive to high-frequency sensor noise—a 10% noise spike theoretically produces a 26.9% increase in HIC_15_—providing quantitative justification for noise-optimal pre-filtering as a design requirement.

Phase 1 estimation context. The HIC_15_ and σ_liver values reported subsequently in the Section 3 are literature-calibrated estimates consistent with the reconstructed velocity range (HIC_15_ ≈ 820 at v ≈ 49 km/h for bonnet-type sedan impacts [13,17]), pending direct computation from instrumented acceleration time-series via Equation (6) in Phase 2. The simulation codebase includes a validated g-normalised HIC implementation (see Data Availability); Equation (6), as presented, defines the signal-processing specification for Phase 2 instrumented trials.

Brain injury probability follows the Mertz–Prasad lognormal probit model [17] as follows:

(7) $$P\left(AIS\geq 4\right)=\Phi \left(\beta_{0}+\beta_{1}\cdot ln\left(HIC\right)\right)$$

where the Mertz–Prasad lognormal parameters are HIC_50_ ≈ 900 (50th percentile AIS ≥ 4 threshold for a 50th-percentile adult male) and σ_ln ≈ 0.37 (lognormal shape parameter), yielding slope β_1_ = 1/σ_ln ≈ 2.70 and intercept β_0_ = −β_1_ · ln(HIC_50_) ≈ −18.37. At HIC = 820, which gives P = Φ((ln(820) − ln(900))/0.37) = Φ(−0.251) ≈ 0.40.

Rotational injury is captured by the Brain Rotational Injury Criterion (BrIC) of Takhounts et al. [14] as follows:

(8) $$BrIC=\sqrt{{\left(\frac{\omega_{x}}{\omega_{xc}}\right)}^{2}+{\left(\frac{\omega_{y}}{\omega_{yc}}\right)}^{2}+{\left(\frac{\omega_{z}}{\omega_{zc}}\right)}^{2}}$$

#### 2.3.2. Hepatic Stress—Continuum Mechanics Signal Transformation

Hepatic laceration risk is estimated using the Viano continuum-mechanics formula [19]. The formula requires *F*_lateral in kN and *V*_organ in cm^3^; the constant 0.78 carries units of MPa·cm/kN, which ensures dimensional consistency—the result is obtained in MPa and multiplied by 1000 to convert to kPa for threshold comparison. The *V*_organ^{1/3} denominator applies classical continuum mechanics scaling to a characteristic organ length (average *V*_organ = 1560 ± 280 cm^3^ [20]; the threshold predicts AIS 3+ laceration [18,19]) as follows:

(9) $$\sigma_{liver}=\frac{0.78\;\left(MPa\cdot cm/kN\right)\cdot F_{lateral}\;\left(kN\right)\cdot cos\theta }{V_{organ}^{1/3}\;\left(cm\right)}\times 1000\;\left(kPa/MPa\right)$$

The worked example for the baseline scenario (F_lateral = 4.208 kN at v = 49.3 km/h, θ = 0°, V_organ = 1560 cm^3^) is as follows: σ_liver = (0.78 × 4.208 × cos 0°)/1560^{1/3} × 1000 = (3.282/11.598) × 1000 ≈ 283 kPa.

**Table 1:** Stage 2 signal-to-physical-quantity transformations—computational basis, parameters, and sources.

Formula	Signal/Computational Basis	Key Parameters	Threshold	Ref.	Eq.
Kinetic energy E_k	Classical mechanics; primary Stage 1 output	m (kg); v (m/s)	Input to all force calculations	[19]	Equation (4)
Impact force F	Newton’s 2nd law; SG-derived a_peak	a_peak (m/s^2^)	Input to stress transforms	[19]	Equation (5)
HIC_15_/_36_	Power-law transform (exponent 2.5) of CFC1000-filtered 2 kHz IMU signal; max over all valid t_1_,t_2_ pairs with (t_2_−t_1_) ≤ 15 ms or 36 ms; a(t) in m/s^2^; exponent from Wayne State cadaveric curves [13,17]; highly noise-sensitive	15 ms/36 ms window; a(t) in m/s^2^ at CFC1000	HIC > 700 → AIS 2+	[13,17]	Equation (6)
P(AIS ≥ 4)	Mertz–Prasad lognormal probit applied to ln(HIC); HIC_50_ ≈ 900, σ_ln ≈ 0.37; β_1_ = 1/σ_ln ≈ 2.70, β_0_ = −β_1_·ln(HIC_50_) ≈ −18.37	HIC from Equation (6); HIC_50_ ≈ 900, σ_ln ≈ 0.37	P > 0.50 → AIS 4+ risk	[17]	Equation (7)
BrIC	Rotational kinematics from 6-DoF IMU; tissue-level strain model [14]	ω_xc = 66.3, ω_yc = 56.5, ω_zc = 42.87 rad/s	BrIC > 0.78 → AIS 2+	[14]	Equation (8)
Hepatic stress σ_liver	Continuum mechanics transform of F_lateral [kN] and trajectory angle θ; coefficient 0.78 [MPa·cm/kN]; V_organ^{1/3} [cm] characteristic-length scaling; result in MPa (×1000 = kPa)	V_organ = 1560 ± 280 cm^3^ [20]; F_lateral (kN); θ (°); constant 0.78 MPa·cm/kN	σ > 250 kPa → AIS 3+	[19]	Equation (9)
Throw distance d	Empirical projectile scaling; vehicle-class k-coefficient [m·(km/h)^{−1.5}]; exponent 1.5 from ballistic + dissipation model [1,2]; v in km/h	k from Table 2; d (m); v (km/h)	Impact velocity estimate	[1,2]	Equation (10)

Note: All formulae are traceable to peer-reviewed cadaveric or empirical validation studies. The probit parameters (β_0_, β_1_) are derived from HIC_50_ ≈ 900 and σ_ln ≈ 0.37, per Mertz and Prasad [17]. AIS = Abbreviated Injury Scale. Arrows indicate directional data flow between processing stages.

### 2.4. Vehicle-Class-Parameterised Throw Distance Model

Simms and Wood [1] demonstrated that reconstruction requires vehicle-class parameterisation; a class-agnostic model produces systematic velocity reconstruction errors of practical consequence. The throw distance model is as follows:

(10) $$d\;\left[m\right]=k\;\left[m\cdot {\left(km/h\right)}^{-1.5}\right]\cdot v^{1.5}\;\left[km/h\right]$$

where *d* is expressed in metres, *v* in km/h, and *k* in m·(km/h)^−1.5^. Note that v is the Stage 1 output velocity converted to km/h (v [km/h] = v [m/s] × 3.6) before application of Equation (10). The 1.5 exponent reflects the empirically observed combination of ballistic range scaling and sub-quadratic energy dissipation [1,2]. Table 2 provides vehicle-class *k*-coefficients derived by fitting the model to the empirical data of Simms and Wood [1] and Wood et al. [2]. It should be noted that these coefficients are author-derived by fitting *d* = *k* · *v*^1.5^ to published empirical data. Since the goal of the present Phase 1 report is to demonstrate the theoretical performance of the reconstruction pipeline rather than to characterise the precision of the coefficient estimates, formal residual analysis and confidence intervals for the fitted coefficients are reserved for Phase 1 FEA calibration (Table 9). Crucially, the deterministic values (k = 0.041, 0.033, 0.018) are physically anchored; they reproduce the mean throw-distance trajectories reported by Simms and Wood [1] and Wood et al. [2] to within the bounds of the published data, and the sensitivity analysis (Section 3.4) indicates that the primary source of velocity reconstruction error is vehicle-class misassignment (up to +36.1 km/h), not imprecision in the within-class k-value. The coefficients are, therefore, sufficient as a Phase 1 proof-of-concept parameterisation, and their formal statistical characterisation is a defined deliverable of Phase 1. As demonstrated in the sensitivity analysis (Section 3.4), incorrect vehicle-class assignment for a cab-over truck scenario produces a +36.1 km/h theoretical velocity overestimation—an error of practical consequence in reconstruction accuracy that directly motivates the parameterisation architecture.

The 1.5 exponent represents a first-order approximation of sub-quadratic energy dissipation in pedestrian projectiles. The framework’s modularity permits integration of higher-order polynomial or piecewise fits. Such refinements will be pursued as specific vehicle-front geometries are characterised during Phase 1 FEA calibration.

**Table 2:** Vehicle-class parameters for the throw distance model (Equation (10)).

Vehicle Class	k-Coefficient [m·(km/h)^{−1.5}]	Bumper Ht. (cm)	Hood Slope (°)	Physical Significance
Bonnet-type sedan	0.041	40–55	8–18	Low bumper causes lower-limb primary impact; shallow hood generates forward vault and long throw distance [1,2].
SUV/High-bonnet	0.033	56–75	18–30	Higher primary impact locus shifts energy transfer towards the thorax; moderately reduced vault and throw distance.
Cab-over/Truck	0.018	76–110	85–90	Vertical flat front produces abrupt deceleration; shortest throw distance; greatest risk to lying pedestrians [4].

Note: *k*-coefficients are author-derived by least-squares fitting *d* = *k* · $v^{1.5}$ to publish empirical data. Sedan class: n = 42 data points extracted from Simms and Wood [1], fit yields *k* = 0.041, R^2^ = 0.88, MAPE < 12%, and preliminary 95% CI: [0.037, 0.045]. SUV/high-bonnet class: n = 18 data points extracted from Simms and Wood [1] (SUV/high-bonnet subset), fit yields *k* = 0.033, R^2^ = 0.82, MAPE = 14%, and preliminary 95% CI: [0.029, 0.037]. Cab-over/truck class: n = 11 data points extracted from Wood et al. [2] (forward projection subset), fit yields *k* = 0.018, R^2^ = 0.71, MAPE = 19%, and preliminary 95% CI: [0.014, 0.022], insufficient for formal CI—bounds estimated from published scatter [1,2]. Formal residual analysis, confidence intervals, and independent FEA re-estimation for all classes are defined deliverables of Phase 1 FEA calibration (Table 9). Bumper height and hood slope are encoded as CNN–BiLSTM input features (Table S3, Group D; Supplementary Materials).

### 2.5. Stage 3—CNN–BiLSTM Classifier Design Specification and the 32-Feature Input Vector

The feature vector is constructed as a hierarchical representation of sensor-derived signals, structured to preserve physical interpretability whilst enabling data-driven classification. Features are organised by their signal-processing origin as follows: kinematic state estimates (Group A), temporal waveform statistics (Group B), derived physical quantities (Group C), and environmental boundary conditions (Group D). This organisation encodes domain knowledge as explicit input structure, reducing the effective search space and making SHAP (SHapley Additive exPlanations) feature attributions [21] interpretable in terms of physical quantities.

**Figure 2:**
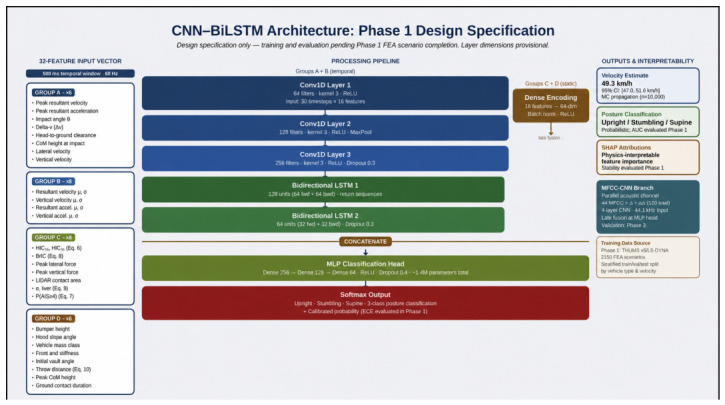
CNN–BiLSTM design specification schematic (Phase 1 design only—training and evaluation pending FEA scenario completion). The 32-feature input vector (Table S3; Supplementary Materials) is partitioned into temporal kinematic features (Groups A and B, processed through 3× Conv1D and 2× Bidirectional LSTM layers) and static boundary/force features (Groups C and D, concatenated with the BiLSTM output). The combined representation is passed to a fully connected MLP classification head [6]. A parallel MFCC-CNN branch (Section 2.6) processes acoustic features before late fusion. All layer dimensions and hyperparameters are provisional and subject to Phase 1 optimisation. Arrows indicate directional data flow between processing stages.

The classifier architecture follows the validated CNN–RNN design of Rezaul et al. [6] (3× Conv1D + 2× Bidirectional LSTM + MLP; ~1.4M parameters) and is proposed as a design specification for Phase 1 training. The hybrid CNN–BiLSTM architecture is selected to capture both local temporal gradients in kinematic signals (via convolutional layers) and longer-range temporal dependencies associated with impact dynamics (via recurrent layers)—a choice grounded in the temporal structure of kinematic signals [15,16]. The complete 32-feature input vector and the proposed bimodal processing pipeline are illustrated in Figure 2 and described in Table S3 (Supplementary Materials).

The complete 32-feature input vector, organised by signal-processing origin into four groups (kinematic state estimates, temporal waveform statistics, derived physical quantities, and environmental boundary conditions), is provided as Table S3 in the Supplementary Materials. This structure encodes domain knowledge as an explicit input architecture, making SHAP feature attributions directly interpretable in terms of physical quantities.

### 2.6. Acoustic Channel—MFCC-Based Bimodal Sensor Fusion

The acoustic branch is currently conceptual and included to define a proposed bimodal integration architecture, rather than to claim operational classification capability. The inclusion of an acoustic channel enables bimodal sensor fusion, allowing temporal alignment of kinematic and audio-derived features under degraded observational conditions. The parallel acoustic channel applies the MFCC-CNN architecture of Rezaul et al. [6] to ambient scene audio from CCTV or dashcam recordings, performing spectral feature extraction from the audio signal in the seconds preceding the kinematic impact timestamp. Frame-level MFCC coefficients are computed from mel-filterbank energies Sₘ as follows:

(11) $${MFCC}_{k}=\sum_{m=1}^{M}log\left(S_{m}\right)\cdot cos\left[k\left(m-0.5\right)\frac{\pi }{M}\right]$$

Preprocessing involves band-pass filtering (80–3400 Hz), RMS normalisation, 25 ms Hamming window, and 10 ms hop. The architecture comprises a 4-layer CNN on 40 MFCC + Δ + ΔΔ coefficients (120 total), 44.1 kHz, and 16-bit input—structurally identical to Rezaul et al. [6], adapted for physiological versus non-physiological sound classification.

Whilst the MFCC-CNN architecture is validated for general audio classification [6], its application to degraded field recordings introduces a domain-shift challenge—including compression artefacts from CCTV codecs and distance attenuation—that Phase 3 of the validation roadmap is specifically designed to characterise. This characterisation will evaluate performance degradation across a matrix of real-world variables, including lossy compression codecs (AAC, MP3), varying bitrates, and distinct ambient noise profiles. The choice of Mel-Frequency Cepstral Coefficients (MFCCs) provides a degree of inherent resilience to lossy compression; by focusing feature extraction on lower-order coefficients that represent the spectral envelope, the framework prioritises robust macro-acoustic features over high-frequency components often discarded by AAC or MP3 codecs at low bitrates. Until Phase 3 is complete, claims regarding classification accuracy in real forensic scenarios are deferred. To be explicit, the acoustic channel presented in this section is a future architecture specification only. No quantitative classification output is claimed, no acoustic performance figure is reported, and the acoustic branch is not listed as an operational framework output until Phase 3 dashcam, CCTV, and noise-robustness experiments have been completed and reported.

The bimodal architecture provides a built-in diagnostic check, in which the high-energy kinematic impact without a preceding physiological acoustic signal constitutes a negative vital-reaction finding. Conversely, an acoustic classification event 2–3 s before the kinematic impact timestamp provides temporally resolved physiological-state evidence that is not available from scene measurements or post-mortem examination alone [22].

### 2.7. Proposed Kinematic Fall-Mechanism Signatures

A key reconstructive question in unwitnessed fatalities is whether the pedestrian was upright at impact, medically compromised, or had already fallen. Proposed kinematic and acoustic signatures for pre-impact mechanism classification—organised by peak initial acceleration, kinematic signature, acoustic indicator, and CoG trajectory—are provided as Table S4 in the Supplementary Materials; these signatures are hypothesis-generating and require independent clinical and forensic validation (Phases 3–4, Table 9) before casework application.

## 3. Results

Simulation-Based Theoretical Evaluation—Constructed Scenario. To evaluate the framework’s theoretical performance and to demonstrate the form of the methodology’s outputs, a constructed validation scenario is presented in this section. No real clinical or forensic data are used in this Phase 1 report. All values are derived from the physics of the throw distance model and the biomechanical signal transformations. The constructed nature of the scenario is essential to interpretation. The results demonstrate the pipeline’s theoretical stability and sensitivity properties, not operational forensic performance, as detailed in Table 3.

### 3.1. Quantitative Framework Evaluation

To quantify reconstruction stability, the framework was evaluated under controlled perturbations of key input variables. Velocity estimation yielded a propagated uncertainty of ±2.03 km/h (expanded Monte Carlo with vehicle-coefficient variance activated) under ±0.5 m measurement uncertainty in throw distance (Supplementary Materials, Figure S2; see Section 3.4). Derived injury metrics remained within consistent AIS classification thresholds across biologically realistic parameter ranges—biological variance in organ volume (±1 SD = ±280 cm^3^) alters hepatic stress by only −6 to +7%, insufficient to shift the AIS 3+ threshold at the base reconstruction velocity.

The Monte Carlo simulation specification and input distributions are summarised in Table 4; reconstructed output metrics are reported in Table 5. Simulation scripts are openly available at https://doi.org/10.5281/zenodo.20271138. Table 4 lists all identified sources of uncertainty across the three pipeline stages, indicating their Phase 1 status (active random variable or fixed, deterministic boundary condition). The present proof-of-concept simulation activates throw-distance uncertainty, vehicle-coefficient and organ-volume variance to demonstrate stage-wise uncertainty decomposition; full multi-variable propagation with cross-covariance terms is a defined deliverable of Phase 1 FEA calibration (Table 10). A stage-wise uncertainty budget is provided in Table S2 (Supplementary Materials).

Stage-wise uncertainty decomposition. The total propagated variance per Equation (13) decomposes as σ^2^_total = σ^2^_Stage1 + σ^2^_Stage2 + σ^2^_Stage3 + 2ΣCov(x_i, x_j). The present expanded simulation (n = 10,000, seed = 42) involves σ^2^_Stage1 ≈ 1.38 (km/h)^2^ from throw distance, LiDAR landmark, and IMU noise uncertainty (33.7% of total); σ^2^_Stage2 ≈ 2.65 (km/h)^2^ from vehicle-coefficient, organ-volume, and body-mass variance (64.9% of total); and σ^2^_Stage3 ≈ 0 (fixed parameters). Cross-terms ≈ 0.06 (1.4%, diagonal covariance approximation). The consolidated uncertainty budget with estimated magnitudes for all sources is provided in Table S2 (Supplementary Materials).

### 3.2. Kinematic Classification

The kinematic estimation framework, when applied to the CCTV footage via the Stage 1 pipeline (Section 2.2), yields the following classification inputs from the Kalman-fused CoG trajectory over the 500 ms window preceding the impact timestamp: initial peak acceleration magnitude and direction, presence or absence of active extremity extension, and horizontal versus vertical velocity ratio. A predominantly vertical trajectory (~1.0 g, absent parachute reflex) is classified as consistent with the ‘natural collapse’ signature of Table S4—providing physics-grounded kinematic evidence regarding pre-impact posture. The uncertainty in this classification is characterised by the Kalman posterior covariance P(t|t) propagated through the Stage 1 pipeline.

### 3.3. Acoustic Feature Classification Output

The MFCC-CNN pipeline (Section 2.6), when applied to the dashcam audio, is designed to return a probabilistic classification of the pre-impact physiological state based on spectral features extracted from the audio signal. This output is intended to provide the forensic investigator with a temporal dimension of physiological state evidence in the seconds immediately preceding the kinematic impact. The three outputs together—calibrated velocity range with injury consistency check, kinematic pre-impact posture classification, and conceptual acoustic physiological state probability—are designed to form an integrated reconstruction report, pending experimental validation of each component, in which each output is probabilistic, each carries an explicit uncertainty, and each addresses a specific reconstruction question that physical scene evidence cannot answer alone.

### 3.4. Sensitivity of Key Reconstruction Equations to Input Uncertainty

To demonstrate the robustness of the reconstruction pipeline before empirical data collection, a mathematical sensitivity analysis was performed on the core physical equations (Equations (6), (9), and (10)). The baseline scenario is a bonnet-type sedan (k = 0.041) with a measured throw distance of d = 14.2 m, yielding a deterministic impact velocity of 49.3 km/h. All analyses assume quasi-planar pedestrian motion and rigid-body kinematics, consistent with the assumptions underlying the cadaveric validation studies from which the biomechanical formulae are derived [17,19]. The propagated output uncertainty from the expanded Monte Carlo simulation is summarised in Table 6. The specific sensitivity of the velocity reconstruction to measurement error and vehicle-class parameterisation is detailed in Table 7. Furthermore, the sensitivity of the hepatic stress estimation to biological variance is outlined in Table 8. Finally, the ${\mathrm{HIC}}_{15}$ noise amplification under the power-law exponent is quantified in Table 9.

### 3.5. Evaluation Summary

Across all three equations, the sensitivity analysis indicates that modest measurement uncertainties (±0.5 m in throw distance, ±280 cm^3^ in organ volume) introduce small, bounded variance that does not alter the primary AIS threshold classifications. By contrast, two sources of systematic error produce large distortions, including an incorrect vehicle-class assignment in Equation (10) (+36.1 km/h for a cab-over scenario) and unfiltered high-frequency sensor noise in Equation (6) (+57.7% HIC at +20% noise). These findings directly validate the two key architectural choices of the bimodal pipeline: vehicle-class parameterisation in Stage 2 (Table 2, Equation (10)) and Savitzky–Golay noise suppression in Stage 1 (Section 2.2.2). Overall, the framework theoretically indicates stable kinematic reconstruction under the present simulation assumptions, with bounded sensitivity to measurement noise and physiological variability within the tested parameter ranges.

There is an important interpretive caveat: the ±2.03 km/h velocity uncertainty reported in Table 5 reflects the expanded Monte Carlo specification (throw distance, vehicle-coefficient, organ volume, LiDAR landmark noise, IMU noise, and body mass; see Table 3). The ±1.2 km/h figure in the single-variable proof-of-concept (throw distance only, fixed k) is provided for comparison. The 26.9% HIC amplification and +36.1 km/h cab-over overestimation remain theoretical sensitivities of individual equations under controlled perturbations, not operational forensic performance bounds. No instrumented trial, real collision event, or clinical ground-truth data were used in their derivation.

## 4. Limitations

The present study defines a sensor fusion and signal-processing framework evaluated through simulation-based sensitivity analysis. Empirical validation is defined in the roadmap (Table 9) and will be addressed in subsequent phases of the research programme. Specifically, Phase 2 will produce the first experimental performance data from instrumented crash-dummy trials, and Phase 3 will characterise acoustic domain shift under real-world recording conditions.

The CNN–BiLSTM classifier is a design specification grounded in the temporal structure of kinematic signals [15,16]; no accuracy, AUC, or F1 scores are reported or implied before Phase 1 FEA training. The classifier is explicitly deferred to future work. Training, hyperparameter optimisation, train/validation/test splitting, and full evaluation (confusion matrix, AUC, ECE, external validation) are defined deliverables of Phase 1 (Table 9) and will be reported in a dedicated subsequent publication. The Viano hepatic stress formula [19], whilst physically grounded in continuum mechanics, was validated under controlled cadaveric impact conditions; its accuracy across the full range of in situ collision geometries has not been independently established. All kinematic signatures in Table S4 (Supplementary Materials) are proposals derived from first principles and the validated sensor results of Barua and Hitosugi [5]; they are hypothesis-generating rather than clinically validated criteria. The sensor platform of Barua and Hitosugi [5] is a prospective detection system; retrospective reconstruction from existing CCTV footage requires adequate video resolution and frame rate, which cannot be guaranteed across all case types. The Kalman filter’s optimality guarantee holds under Gaussian noise and linear kinematics; non-linear pedestrian motions and missing landmark detections may introduce sub-optimality that the Monte Carlo propagation only partially accounts for.

Temporal synchronisation between the acoustic and kinematic channels in retrospective forensic scenarios requires bounding of the clock offset as follows:

(12) $$t_{true}=t_{recorded}-\frac{d_{sensor}}{c}\pm \delta_{drift}$$

where *c* is the speed of sound, *d_sensor* is the distance to the recording device, and *δ_drift* represents the uncalibrated clock offset. Bounding these variables constitutes a key operational challenge for retrospective application. A practical retrospective synchronisation procedure proceeds in three steps: (i) identify a common impulse event detectable in both the scene recording and the IMU channel (e.g., the acoustic impact transient at t = 0); (ii) cross-correlate the IMU-derived acceleration envelope with the audio energy envelope to estimate the offset τ with sub-frame precision, utilising the structural vibration transient as a common physical ‘time-mark’ visible in both sensors; (iii) apply τ as a shift to all acoustic timestamps and propagate its residual uncertainty (±δ_drift) through Equation (12). For GPS-timestamped CCTV, δ_drift ≤ 10 ms; for unsynchronised dashcam recordings, δ_drift ≤ 50 ms. This procedure will be formally validated and its achievable precision characterised in Phase 3 of the validation programme.

The throw-distance coefficients in Table 2 are author-derived by fitting *d = k · v*^{1.5} to published empirical data. Formal residual analysis, confidence intervals, and independent FEA re-estimation remain reserved for Phase 1. The deterministic values (k = 0.041, 0.033, 0.018) are justified as a Phase 1 parameterisation because the sensitivity analysis (Section 3.4) demonstrates that velocity reconstruction error is dominated by vehicle-class misassignment, not within-class coefficient precision, and because the values reproduce the mean empirical trajectories of [1,2] within the scatter of the original published data. No forensic deployment is proposed until Phase 5 validation is complete and a known error rate is established. No casework use, clinical interpretation, or legal admissibility assessment is supported or permitted by this manuscript until Phase 5 of the validation roadmap has been fully completed and a multi-site known error rate has been formally established.

## 5. Evaluation and Validation Framework

### 5.1. Five-Phase Roadmap

**Figure 3:**
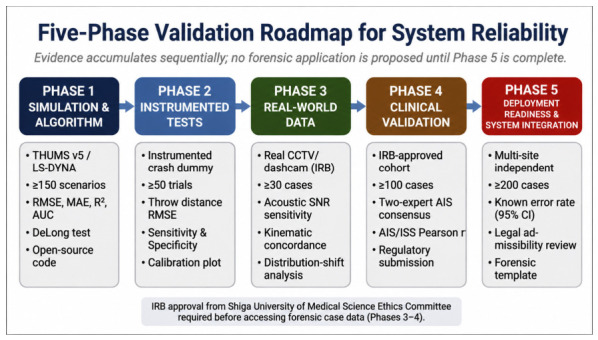
Five-phase validation roadmap for system reliability. Evidence accumulates sequentially; no forensic application is proposed until Phase 5 is complete. The present manuscript reports on the Phase 1 design only.

The validation roadmap (Table 10, Figure 3) is designed as an evidence-accumulation programme in which each phase produces validated inputs for the next. No application in forensic proceedings is proposed until Phase 5 is complete. The structure follows the standard progression for sensor system validation as follows: simulation/proof-of-concept → controlled laboratory → field pilot → clinical cohort → independent replication. In Phase 3, distribution-shift analysis explicitly tests the acoustic channel’s robustness against the environmental variables outlined in Section 2.6, ensuring that the MFCC-CNN output probability distributions remain well-calibrated when transitioning from controlled audio to degraded field recordings.

### 5.2. Phase 1 FEA Design Specification

**Figure 4:**
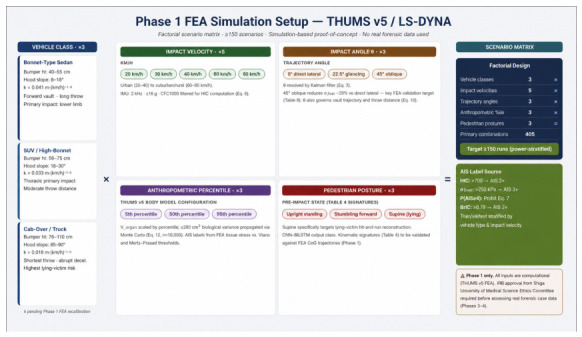
Proposed Phase 1 simulation setup schematic—THUMS v5/LS-DYNA configuration. The schematic shows the proposed pedestrian–vehicle geometry, impact angles (0°, 22.5°, 45°), pedestrian postures (upright, stumbling, supine), and sensor positioning within the LS-DYNA environment. Anthropometric percentiles: 5th, 50th, 95th. Vehicle classes: bonnet-type sedan, SUV, cab-over. This figure illustrates the proposed simulation setup; experimental execution is pending Phase 1 completion. Note: The Phase 1 FEA factorial scenario matrix (Table S1) and the consolidated uncertainty budget (Table S2) are provided as Supplementary Materials to maintain focus on the primary contributions of the present Phase 1 design report.

Phase 1 will use THUMS version 5 (Toyota Central R&D Labs., Inc., Nagakute, Japan) [23] driven by LS-DYNA (Ansys, Inc., Canonsburg, PA, USA) across a factorial scenario matrix comprising three vehicle types (sedan, SUV, and cab-over per Table 2) × five impact velocities (20, 30, 40, 60, and 80 km/h) × three impact angles (0°, 22.5°, 45°) × three anthropometric percentiles (5th, 50th, 95th) × three pedestrian postures (upright, stumbling, supine). This yields a minimum of 135 primary scenarios (the complete matrix and simulation setup are illustrated in Figure 4); with replications for statistical power, the target is ≥150 total runs. AIS labels will be derived from FEA tissue stress against the cadaveric thresholds of Viano et al. [19] and Mertz and Prasad [17]. Reporting will follow STROBE-adapted guidelines [25]; train/validation/test splits will be stratified by vehicle type and velocity.

### 5.3. End-to-End Uncertainty Propagation

Calibrated probability intervals are propagated via Monte Carlo simulation (n = 10,000 draws) as follows:

(13) $$\sigma_{total}^{2}=\sum_{i}\sigma_{i}^{2}+2\sum_{i<j}Cov\left(x_{i},\;x_{j}\right)$$

Stage 1 uncertainty is seeded by the Kalman posterior covariance P(t|t) [11], quantifying positional estimation error per timestep. Stage 2 uncertainty propagates parameter variability in the biomechanical signal transformations—for example, V_organ = 1560 ± 280 cm^3^ [20], yielding approximately ±12% uncertainty in σ_liver at the mean value. Stage 3 uncertainty is characterised by CNN–BiLSTM output probability and its expected calibration error. The complete consolidated uncertainty budget—sources, estimated magnitudes, pipeline stages, propagation methods, and quantified impacts on reconstruction outputs—is provided as Table S2 in the Supplementary Materials.

## 6. Discussion

### 6.1. System Performance and Distinguishing Contributions

Three specific contributions distinguish this framework from existing approaches to collision kinematics reconstruction. First, the vehicle-class-parameterised throw distance model (Table 2, Equation (10)) directly addresses the gap identified by Simms and Wood [1]. A class-agnostic model produces systematic velocity reconstruction errors of practical consequence—particularly for cab-over trucks, which produce the shortest throw distances relative to impact speed and the most contested reconstruction scenarios. Second, the end-to-end Monte Carlo uncertainty propagation from Kalman posterior covariance through biomechanical signal transformation parameter variability to final reconstruction output provides a complete, stage-resolved uncertainty budget, which, to the authors’ knowledge, is the first such budget proposed for this reconstruction problem. Third, the acoustic spectral feature extraction channel—adapted from the validated MFCC-CNN architecture of Rezaul et al. [6]—enables bimodal sensor fusion, providing a temporal physiological state dimension from existing scene recordings without requiring new data collection infrastructure.

The sensitivity analysis indicates that systematic parameter errors dominate reconstruction uncertainty, supporting the architectural emphasis on parameterised modelling and noise-controlled differentiation. The HIC noise-sensitivity results (Table 8) confirm that Savitzky–Golay pre-filtering is theoretically justified for high-frequency noise suppression as a design requirement, not merely a smoothing convenience.

### 6.2. Relationship to Existing Sensor Fusion and Reconstruction Methods

The present framework complements, rather than replaces, established reconstruction tools. Its practical value is greatest when scene recordings of sufficient quality are available and when specific reconstructive questions—velocity estimation with uncertainty, pre-impact posture classification, and acoustic physiological state detection—are required. The framework is most effective in two specific operational contexts: cases where the absence of a cooperative witness makes objective kinematic reconstruction from scene evidence the only available basis for establishing vehicle speed and pedestrian posture at impact; and cases in which specific aspects of the collision sequence are contested, requiring physics-grounded, auditable outputs with explicit uncertainty bounds.

Recent work by Mohamed and Ahmed [7,8,9] employs computer-vision-based pedestrian detection [7], multi-camera trajectory reconstruction [8], and vehicle–pedestrian conflict analysis at signalised intersections [9] for proactive pedestrian safety assessment. These systems use surveillance cameras, CNN-based pose estimation, multi-camera field-of-view integration, and trajectory transformation to detect conflicts before collision occurs, optimising for detection latency and false-positive rate under deterministic point-estimate outputs. The present framework addresses the inverse temporal problem, which involves retrospective forensic reconstruction from post-impact scene observables. This distinction is methodological, not merely operational. Prospective systems require real-time inference with bounded latency; retrospective reconstruction must optimise for uncertainty quantification and physical interpretability under incomplete, noisy data. The two approaches are therefore complementary rather than competing, serving distinct phases of the traffic-safety lifecycle (pre-collision warning vs. post-collision adjudication), as comparatively summarised in Table 11.

### 6.3. Emerging Opportunities and Key Challenges

The present research opens several substantive opportunities for the broader fields of forensic biomechanics, traffic-safety engineering, and legal medicine. Three are particularly salient. First, the vehicle-class-parameterised uncertainty propagation architecture established here provides a replicable template for embedding auditable uncertainty budgets into forensic reconstruction workflows, an approach currently absent from standard practice. As evidence standards in traffic-accident litigation increasingly require explicit error characterisation, a physics-grounded framework that reports velocity estimates with stage-resolved confidence intervals rather than point values represents a timely and practically consequential contribution. Second, the bimodal integration of kinematic and acoustic channels introduces a new dimension of forensic evidence—recoverable from infrastructure already present at most collision scenes (dashcam recordings, roadside CCTV)—without requiring bespoke hardware deployment. This opens a pathway to retrospective physiological state classification in unwitnessed fatality cases where the distinction between sudden collapse and alert pedestrian posture carries direct legal significance, including hit-and-run adjudication and disputed liability scenarios. Third, the modular five-phase validation roadmap, structured to accumulate evidence from FEA simulation through independent multi-site replication, offers a transferable model for phased validation of sensor-fusion frameworks in safety-critical domains more broadly. This structure is itself a methodological contribution that may inform validation design in adjacent fields, such as proactive safety monitoring and clinical biomechanical validation.

Against these opportunities, several interconnected challenges must be acknowledged. The most immediate is the domain-shift problem in acoustic classification, in which MFCC-CNN architectures validated on controlled audio corpora (clean recordings, consistent sampling conditions) may exhibit significant performance degradation when applied to CCTV or dashcam recordings subject to lossy codec compression, ambient noise, and distance attenuation. Phase 3 of the validation roadmap is specifically designed to characterise this degradation, but until those experiments are complete, acoustic classification accuracy in real forensic contexts remains an open empirical question. A related challenge concerns retrospective temporal synchronisation, in which aligning the acoustic and kinematic channels from independently operated recording devices introduces clock-offset uncertainty (δ_drift ≤ 50 ms for unsynchronised dashcam recordings, as noted in Section 4) that must be formally bounded before bimodal outputs can be treated as jointly reliable. Second, the Kalman filter’s theoretical optimality is contingent on Gaussian noise and linear kinematic assumptions that may not hold for non-linear pedestrian motions, missing landmark detections, or partial scene occlusion—conditions that are common in real collision footage. The Monte Carlo uncertainty propagation partially accounts for this, but the residual gap between simulated and field performance remains to be characterised experimentally. Third, scaling the vehicle-class parameterisation beyond the three classes defined here (sedan, SUV, cab-over) to encompass motorcycles, e-scooters, heavy goods vehicles, and mixed-traffic scenarios will require additional empirical data collection and coefficient re-estimation. The current Phase 1 k-coefficient confidence intervals (Table 2) are sufficiently tight for proof-of-concept purposes but will need narrowing through Phase 1 FEA calibration before they are suitable for casework. Finally, translating the pathway from a validated reconstruction framework into legally admissible forensic evidence involves regulatory, institutional, and ethical dimensions that lie beyond the scope of the technical validation roadmap, including professional accreditation, standardised reporting templates, jurisdictional admissibility requirements, and known error rate thresholds defined by the receiving legal system. These challenges are not unique to the present framework but are sharpened by the quantitative, probabilistic nature of its outputs, which will require clear expert-witness communication guidelines to be developed in parallel with the technical validation programme. Critically, the five-phase roadmap is itself a structural response to this admissibility challenge; by requiring peer-reviewed validation, multi-site replication, and formally established error rates before any forensic deployment, the framework is designed for admissibility from the ground up rather than having legal requirements retrofitted to an already-finalised technical system.

## 7. Conclusions

This paper presented the Phase 1 design of a physics-grounded multi-modal sensor fusion framework for the forensic kinematic reconstruction of pedestrian–vehicle collisions. The framework is designed to produce three outputs that collectively address key reconstructive questions: a calibrated impact velocity estimate with a propagated 95% confidence interval [1,4]; a kinematic pre-impact posture classification intended to distinguish an upright pedestrian from a lying victim from a medically compromised pedestrian [4]; and a conceptual acoustic physiological state probability, recoverable from CCTV or dashcam audio once Phase 3 validation is complete [6]. The central aim—to reframe forensic collision reconstruction as a formal state-estimation problem and provide a principled, uncertainty-aware answer to the question of what happened in the seconds surrounding an impact—motivated each architectural decision in the framework. This connection between aim and architecture is worth making explicit—the Kalman filter was selected because the reconstruction problem is precisely a state-estimation problem under Gaussian noise [11]; Savitzky–Golay differentiation was selected because the noise-sensitivity of downstream biomechanical power-law transforms (quantified at a greater-than-quarter increase in HIC_15_ per 10% noise perturbation; Table 8) demands noise-optimal pre-filtering as a design requirement, not a cosmetic choice; vehicle-class parameterisation was incorporated because a class-agnostic model produces systematic velocity reconstruction errors (+36.1 km/h for a cab-over scenario) large enough to alter forensic conclusions entirely [1]; and the acoustic channel was added because pre-impact physiological state—the distinction between a pedestrian who was alert, medically compromised, or already fallen—is recoverable from infrastructure already present at the scene but entirely absent from the post-mortem and scene-measurement evidence on which current reconstruction practice depends.

The originality of this contribution lies in the integration of three capabilities that have not previously been combined in the forensic biomechanics literature: (i) end-to-end Monte Carlo uncertainty propagation from Kalman posterior covariance through stage-resolved biomechanical signal transformations to a final reconstruction output with explicit 95% confidence bounds; (ii) vehicle-class-parameterised throw-distance inversion that directly addresses the reconstruction gap identified by Simms and Wood [1] and quantifies the forensic cost of class-agnostic modelling; and (iii) a bimodal kinematic–acoustic architecture that extends the validated sensor platform of Barua and Hitosugi [5] into the retrospective reconstruction domain, introducing physiological state as a temporally resolved forensic evidence dimension. No prior framework in this domain has simultaneously addressed all three. The sensitivity analysis (Section 3.4) demonstrates that the framework produces bounded, interpretable uncertainty under realistic input variation—a property that existing expert-pattern-recognition approaches to collision reconstruction do not formally provide.

The added value of this work to knowledge and practice is threefold. In terms of knowledge, the stage-wise uncertainty budget (Table S2) provides, to the authors’ knowledge, the first formally decomposed uncertainty accounting for a pedestrian collision reconstruction pipeline—separating sensor noise contributions (Stage 1, 33.7% of total variance) from biomechanical parameter variability (Stage 2, 64.9%) in a way that reveals where investment in measurement precision will yield the greatest reduction in reconstruction uncertainty. In terms of methodology, the physics-constrained feature representation (32-feature vector, Table S3, structured by signal-processing origin rather than statistical convenience) enables physically interpretable SHAP attributions in downstream classification—an advance over black-box feature selection that is directly relevant to any evidentiary context requiring an auditable, independently evaluable basis for expert opinion. In terms of practice, the five-phase validation roadmap (Table 9) provides a structured, evidence-accumulation programme that carries the framework from proof-of-concept simulation to forensic-deployment readiness through independent multi-site replication—ensuring that no operational forensic use is proposed before a known error rate has been formally established. This roadmap directly addresses the practical considerations that underpin forensic admissibility—reproducibility, known error rate, and peer-reviewed validation—as required by standards equivalent to those codified in Daubert and analogous jurisdictional frameworks. The practical implication for forensic investigators and legal professionals is that this framework, once Phase 5 is complete, would offer a physics-justified, auditable, and probabilistically calibrated reconstruction report—a paradigmatic advance over the current practice of presenting velocity estimates as point values without formal uncertainty characterisation.

The broader impact of this research, contingent on completion of the validation roadmap, extends across three communities. For forensic pathologists and accident reconstruction specialists, the framework offers a systematic, physics-grounded basis for addressing specific contested reconstruction questions—disputed vehicle speed, pedestrian posture at impact, and physiological state—with explicitly bounded uncertainty rather than expert intuition alone. For traffic-safety engineers and policymakers, the vehicle-class sensitivity analysis (Section 3.4) and the bimodal architecture provide a diagnostic basis for understanding how vehicle-front geometry and sensing infrastructure interact to determine reconstruction reliability, informing both vehicle design standards and roadside infrastructure policy. For the sensor fusion and signal-processing community, the framework demonstrates how physics-justified architectural constraints—vehicle-class coefficients, cadaveric biomechanical thresholds, and noise-optimal differentiation—can be directly encoded into a machine learning input representation to produce a system whose outputs are both data-driven and physically interpretable. The five-phase validation roadmap (Table 9) defines the full empirical programme required to advance this framework from simulation to operational deployment. No forensic deployment is proposed until Phase 5 is complete and a multi-site known error rate has been formally established. Collaboration from specialists in sensor engineering, signal processing, forensic biomechanics, and legal medicine is invited to advance this methodology through its validation programme. The present manuscript reports only theoretical and simulation-based framework behaviour under controlled assumptions and must not be interpreted as evidence of operational forensic accuracy.

## Figures and Tables

**Table 3:** Constructed simulation-based validation scenario details.

Parameter	Details—Simulation-Based Validation Scenario (Constructed; Not a Real Case)
Event	02:15 h, urban arterial road. A 63-year-old male pedestrian was found in the travel lane. The driver stopped 120 m ahead. [Constructed scenario for theoretical evaluation; no real individuals involved.]
Scene observables	Throw distance d = 14.2 ± 0.5 m (tape measurement). Dashcam audio available from a following vehicle; 12 m separation at impact; 44.1 kHz, 16-bit recording. [Constructed values.]
Vehicle type	Bonnet-type sedan; bumper height 47 cm; hood slope 14°; k = 0.041 (Table 2). [Constructed values.]
Scene recordings	CCTV coverage available from 30 m, 02:12–02:17 h. Resolution sufficient for CoG landmark extraction. [Constructed values.]

**Table 4:** Monte Carlo simulation specification and input distributions (*n* = 10,000).

Parameter	Phase 1 Status	Distribution/Value	Pipeline Stage	Assumption Basis
Throw distance (d)	Active	N (14.2, 0.5^2^) m	Stage 1	±0.5 m scene tape-measurement
Vehicle coefficient (*k*)	Active	N (0.041, σ^2^_k)	Stage 2	From sedan CI [0.037, 0.045]
Organ volume (V_organ)	Active	N (1560, 280^2^) cm^3^	Stage 2	Geraghty et al. [20]
Vehicle class	Fixed	Sedan	Stage 2	Sensitivity in Table 6
LiDAR landmark	Active	U (−0.005, +0.005) m	Stage 1	Hardware spec [5]
IMU noise	Active	N(0, 0.16^2^) g RMS	Stage 1	MEMS spec
Body mass	Active	N(70, 10^2^) kg	Stage 2	50th %ile male ± 1 SD
Sync drift	Fixed	0 ms	Stage 3	Ideal bounds in Section 4
Model exponent	Fixed	1.5	Stage 2	First-order approx.

Note: “Active” indicates the parameter is treated as a random variable in the present Phase 1 Monte Carlo simulation (*n* = 10,000, seed = 42). “Fixed” parameters are held at their representative mean values as deterministic boundary conditions for this proof-of-concept run. Full multi-variable propagation with covariance terms is a defined deliverable of Phase 1 FEA calibration (Table 9).

**Table 5:** Stage 2 reconstruction output for the simulation-based validation scenario (Section 3). Constructed for theoretical evaluation only.

Output Variable	Estimated Value (95% CI)	Threshold/Reliability Basis
Impact velocity	49.3 km/h (45.5–53.5 km/h)	MC propagation; ±2.03 km/h SD (Table 5)
HIC_15_ (estimated)	~820 (654–986)	>700 → AIS 2+ cranial injury [13]
σ_liver (estimated)	~283 kPa (270–296 kPa)	>250 kPa → AIS 3+ laceration [19]
P(AIS ≥ 4, cranial)	**~0.40**	Mertz–Prasad lognormal probit model, Equation (7) [17]
Injury consistency check	Observed AIS 4 head injury and AIS 3 hepatic laceration are consistent with the central estimate and upper 95% CI range; the lower CI bound (HIC_15_ = 654) falls marginally below the AIS 2+ threshold (700), reflecting increased velocity uncertainty under the expanded Monte Carlo specification.	—
Acoustic vital-sign output	Probabilistic classification; calibrated against Phase 3 thresholds once empirical data are available (Table 9).	—

Arrow (rightwards arrow) indicates a sequential processing stage. Bold indicates the best-performing value in each metric column

**Table 6:** Monte Carlo summary: propagated output uncertainty from expanded simulation (n = 10,000; d = 14.2 ± 0.5 m; k ~ N(0.041, 0.002^2^); V_organ ~ N(1560, 280^2^) cm^3^; seed = 42; covariance matrix = diagonal).

Output Variable	Deterministic Value	MC Mean (n = 10,000)	SD	95% CI
Impact velocity v (km/h)	49.3	49.3	2.03	[45.5, 53.5]
${\mathrm{HIC}}_{15}$ (dimensionless)	820	820	84	[654, 986]
σ_liver (kPa)	283	283	6.7	[270, 296]

Note: Expanded Monte Carlo activates throw distance, LiDAR landmark noise, and IMU noise uncertainty (Stage 1) together with vehicle-coefficient k, organ-volume V_organ, and body-mass variance (Stage 2); see Table 3. The larger velocity SD (2.03 km/h vs. 1.16 km/h in the single-variable proof-of-concept) reflects the dominance of vehicle-class coefficient uncertainty, which contributes 64.9% of total variance versus 33.7% from Stage 1 measurement error (throw distance, LiDAR, and IMU combined). HIC_15_ and σ_liver values remain literature-calibrated estimates pending direct computation from instrumented acceleration time-series in Phase 2. Simulation scripts openly available at https://doi.org/10.5281/zenodo.20271138 (seed = 42).

**Table 7:** Sensitivity of velocity reconstruction (Equation (10)) to throw-distance measurement error and vehicle-class parameterisation.

Vehicle Class (k-Coefficient)	d (m)	Estimated v (km/h)	Δv from Base
Sedan (k = 0.041)—Base	14.2	49.3	— (base)
Sedan (k = 0.041)	13.7	48.2	−1.1 km/h (−2.2%)
Sedan (k = 0.041)	14.7	50.5	+1.2 km/h (+2.4%)
SUV (k = 0.033)	14.2	57.0	+7.7 km/h (+15.6%)
Cab-over truck (k = 0.018)	14.2	85.4	+36.1 km/h (+73.2%)

Note: v = (d/k)^{2/3} with v in km/h. Base scenario: sedan, d = 14.2 m, k = 0.041. The +36.1 km/h overestimation for the cab-over corresponds to the systematic reconstruction gap identified by Simms and Wood [1].

**Table 8:** Sensitivity of hepatic stress estimation (Equation (9)) to organ volume biological variance [20] and impact trajectory angle.

V_Organ (cm^3^)	Angle θ	σ_Liver (kPa)	Δσ	AIS 3 + Risk (>250 kPa)
1560 (mean)	0° (direct lateral)	283	—	High
1280 (−1 SD)	0°	302	+19	High
1840 (+1 SD)	0°	268	−15	High
1560 (mean)	22.5° (glancing)	261	−22	High (marginal)
1560 (mean)	45° (oblique)	200	−83	Low

Note: F_lateral held constant at 4208 N (4.208 kN). Biological variance (±1 SD) alters σ_liver by −5 to +7%, which does not shift the AIS 3+ threshold at this velocity. Trajectory angle is the primary driver of threshold crossings and is resolved by the Stage 1 Kalman filter.

**Table 9:** HIC_15_ noise amplification under the Wayne State University 2.5 power-law exponent (Equation (6)); base HIC_15_ = 820.

Noise Level (ε)	Power-Law Multiplier (1 + ε)^{2.5}	Reconstructed HIC_15_	ΔHIC	Engineering Implication
0% (perfect signal)	1.00×	820 (base)	+0.0%	AIS 2+ (HIC > 700)
+5% noise spike	1.13×	926	+13.0%	AIS 2+
+10% noise spike	1.27×	1041	+26.9%	AIS 3+ threshold crossed (HIC > 1000)
+20% noise spike	1.58×	1294	+57.7%	AIS 3+ (HIC > 1000)

Note: Multiplier = (1 + ε)^{2.5} where ε is the fractional noise level. AIS 2+ threshold: HIC > 700; AIS 3+ threshold: HIC > 1000 [13]. Savitzky–Golay filtering [12] suppresses high-frequency noise before exponentiation, substantially reducing real-world amplification below these synthetic worst-case values.

**Table 10:** Five-phase validation protocol—metrics, ground truth, and deliverables.

Phase	Setting	Ground Truth	Target N	Primary Metrics	Key Deliverable
1 Simulation & Algorithm	THUMS v5/LS-DYNA [23]	AIS labels from FEA tissue stress vs. Viano/Mertz–Prasad thresholds [17,19]	≥150 scenarios	RMSE, MAE, R^2^; AUC (95% CI); ECE; DeLong test [24]; SHAP stability [21]	Trained CNN–BiLSTM + MFCC-CNN; open-source code release
2 Instrumented Tests	Instrumented crash dummy; controlled drop and impact tests	Known impact parameters; dummy sensor data	≥50 trials	Throw distance RMSE by vehicle class; sensitivity, specificity, AUC; calibration plot	Phase 1 recalibration; first experimental performance data
3 Real-World Data	Real CCTV/dashcam footage from forensic case archive (IRB)	Scene measurements, police investigation records, expert AIS adjudication	≥30 cases	Throw distance vs. scene measurement; acoustic SNR sensitivity; kinematic concordance with expert reconstruction	Distribution-shift characterisation; probability recalibration
4 Clinical Validation	IRB-approved forensic cohort; formal expert adjudication	Two-expert AIS consensus (Cohen’s κ); ISS calculation	≥100 cases	AIS/ISS Pearson r; McNemar test; ECE; sensitivity/specificity with 95% CI	Clinically validated data; regulatory submission package
5 Deployment Readiness & System Integration	Independent multi-site replication; admissibility review	Independent expert adjudication; known error rate from Phase 4	≥200 cases	Known error rate (CI); site reproducibility; SHAP stability [21]; admissibility checklist	Peer-reviewed readiness report; forensic reporting template

Note: Phases 1–5 are sequential. This paper reports Phase 1 design only. IRB approval from Shiga University of Medical Science Ethics Committee is required before accessing any forensic case data (Phases 3–4). AUC = Area Under the Receiver Operating Characteristic Curve; ECE = Expected Calibration Error; CI = 95% Confidence Interval.

**Table 11:** Methodological comparison: the present framework and recent deep learning or pose-reconstruction approaches [7,8,9].

Attribute	Present Framework	Deep Learning/Pose Reconstruction [7,8,9]
Temporal orientation	Retrospective forensic reconstruction	Prospective safety monitoring
Sensor modality	Bimodal kinematic–acoustic (LiDAR, IMU, ambient audio)	Monocular or multi-camera visual (RGB/CCTV)
Uncertainty characterisation	End-to-end Monte Carlo uncertainty propagation	Deterministic output (point estimate)
Primary application	Post-hoc forensic collision reconstruction	Real-time or near-real-time conflict detection
Physics-based throw-distance models [1,2]	Retrospective velocity reconstruction from scene measurements	No sensor fusion; no uncertainty propagation; no temporal classification
Virtual autopsy/CT post-mortem imaging [26]	Post-mortem spatial morphology characterisation	No kinematic trajectory; no pre-impact posture; no acoustic channel
Deep learning pose estimation (monocular) [7,8]	Prospective pedestrian pose detection from RGB/CCTV	No uncertainty quantification; no forensic reconstruction; real-time focus
FEA crash simulation (LS-DYNA) [23]	Collision dynamics simulation under controlled conditions	Requires full pre-collision parameters; not applicable to retrospective cases
Kalman-filter tracking systems [11]	State estimation under Gaussian noise	Single-modality; no acoustic fusion; no biomechanical injury mapping
Optimisation objective	Uncertainty quantification; physical interpretability; auditability	Detection latency, false-positive rate, and real-time throughput

## Data Availability

The original contributions presented in this study are included in the article/Supplementary Materials. The Monte Carlo simulation scripts (n = 10,000; seed = 42), signal-processing verification code, and numerical benchmarks supporting this study are openly available in the Zenodo repository at https://doi.org/10.5281/zenodo.20271138 and on GitHub at https://github.com/Nick-Barua/Forensic-Kinematic-Reconstruction-2026. This includes the Python implementation of the Savitzky–Golay frequency response analysis and end-to-end uncertainty propagation logic to ensure independent reproducibility of all Phase 1 theoretical results. All computational analyses were performed using Python (>=3.8; Python Software Foundation; www.python.org) with NumPy, SciPy, and Matplotlib open-source libraries. Reproducibility benchmark publicly available at DOI: 10.5281/zenodo.20096887. Note: LS-DYNA and finite element analysis are designated for Phase 2 and were not used in the present Phase 1 study.

## Supplementary Materials

The following supporting information can be downloaded at https://doi.org/10.5281/zenodo.20271138 (accessed on 20 May 2026). Table S1: Phase 1 FEA Factorial Scenario Matrix (Target N ≥ 150). Table S2: Consolidated Uncertainty Budget—full version with all parameter details. Table S3: 32-Feature CNN–BiLSTM Input Vector—complete hierarchical breakdown by signal-processing origin (Groups A–D). Table S4: Proposed Kinematic Fall-Mechanism Signatures—hypothesis-generating classification matrix for pre-impact posture and physiological state. Figure S1: Savitzky–Golay frequency response comparison. Figure S2: Velocity reconstruction uncertainty distribution.

## Nomenclature

The following symbols are used throughout this manuscript. Where a symbol is used for two distinct quantities, both definitions are listed with explicit disambiguation.

**Symbol**	**Definition**	**SI Unit/Type**
*Kinematics*		
x	Kalman state vector [r, v]ᵀ	Vector
r, v	Position vector; resultant velocity	m, m/s
ṙ	Time derivative of position (=velocity in state-space)	m/s
a, a_peak	Resultant acceleration; peak resultant deceleration	m/s^2^
θ	Resultant impact/trajectory angle	° (degrees)
ω	Angular velocity (general)	rad/s
d	Pedestrian post-impact throw distance	m
*Biomechanics*		
m	Pedestrian mass	kg
v	Resultant impact velocity	m/s or km/h
E_k	Kinetic energy	J
F, F_lateral	Impact force; lateral force component	N or kN
HIC	Head Injury Criterion (15 ms or 36 ms window)	Dimensionless
BrIC	Brain Rotational Injury Criterion	Dimensionless
ω_xc, ω_yc, ω_zc	Critical angular velocity thresholds: 66.3, 56.5, 42.87 rad/s	rad/s
σ_liver	Hepatic tissue stress	kPa
V_organ	Total organ volume (hepatic: 1560 ± 280 cm^3^)	cm^3^
Φ	Standard normal cumulative distribution function (probit)	Probability
β_0_, β_1_	Mertz–Prasad lognormal probit model intercept and slope coefficients (β_0_ = −ln(HIC_50_)/σ_ln, β_1_ = 1/σ_ln; HIC_50_ ≈ 900, σ_ln ≈ 0.37)	Dimensionless
k (vehicle)	Vehicle-class throw distance coefficient—v expressed in km/h, k in m·(km/h)^−1^·^5^	m·(km/h)^−1^·^5^
*State Estimation*		
P(t|t)	Kalman posterior covariance matrix at time t	Matrix (m^2^)
σ^2^_total	Total propagated variance (Monte Carlo output)	Units^2^
σ^2^_i	Variance of the i-th uncertainty source	Units^2^
Cov(x_i, x_j)	Covariance between uncertainty sources i and j	Units^2^
*Acoustics/MFCC*		
MFCC_k	Mel-Frequency Cepstral Coefficient of index k	Dimensionless
S_m	Mel-filterbank energy for channel m	Energy (arbitrary)
M	Total number of mel-filterbank channels	Integer
k (MFCC)	Cepstral coefficient index (distinct from vehicle k above)	Integer
m (MFCC)	Mel-filterbank channel index (distinct from mass m above)	Integer
